# The neuromechanical delay of the quadriceps shortens with increasing contraction intensity

**DOI:** 10.1038/s41598-025-10477-1

**Published:** 2025-07-14

**Authors:** Rafael Krätschmer, Maleen Stingl, Denis Holzer, Florian Kurt Paternoster

**Affiliations:** 1 Professorship of Biomechanics in Sports, Department Health and Sport Sciences, TUM School of Medicine and Health, Technical University of Munich, Munich, Germany; 2 Prevention Center, Department Health and Sport Sciences, TUM School of Medicine and Health, Technical University of Munich, Munich, Germany; 3 Institute of Sport Science, Department of Human Sciences, Universität der Bundeswehr München, Neubiberg, Germany

**Keywords:** Neuromechanical delay, Neural drive, Force fluctuation, Motor unit, HDEMG, Electromyography - EMG, Motor control

## Abstract

Neuromechanical delay (NMD) quantifies the time lag between the neural drive to a muscle and muscle force production. While NMD varies with factors like contraction speed and muscle group, its response to different contraction intensities remains unclear. This study examined NMD in the quadriceps during isometric trapezoidal contractions at 10%, 30%, 50%, and 70% of maximum voluntary contraction (MVC) in 13 recreationally trained males (24.3 ± 2.8 years). High-density surface electromyography recorded motor unit firings in the vastus lateralis and medialis, with neural drive quantified via cumulative motor unit firings. NMD was determined by cross-correlating neural drive with force signals during steady-force phases. Results showed a significant NMD reduction (*p* < 0.001) with increasing contraction intensity, decreasing by 31% from 175.8 ms (± 44.1) at 10% MVC to 121.6 ms (± 34.6) at 70% MVC. This likely reflects changes in muscle mechanical properties due to higher-threshold motor unit recruitment. These findings enhance our understanding of neuromechanical coupling, revealing a force-dependent modulation of NMD that may have implications for neuromuscular function in health and disease.

## Introduction

Muscle force generation in response to neural drive is not instantaneous; rather, a latency exists between neural activation and muscle force production. The concept of electromechanical delay describes the latency between muscle activation and muscle force generation^1–3^. However, electromechanical delay is limited to measuring the delay at the onset of muscle force from a relaxed muscle state, making it unsuitable for assessing the delay between neural output and muscle force production during ongoing muscle contractions. In contrast, neuromechanical delay (NMD) refers to the time lag between the occurrence of motor unit firings at the muscle evoked by the central nervous system (CNS) and the muscle force generation during sustained contractions^4^. NMD is quantified as the delay between the neural drive to the muscle, represented by the cumulative output of active motor units (MUs) during a muscle contraction^5^, and the resulting muscle force; it is measured by cross-correlating the neural and the force signal, identifying the time lag between neural output and force output^4^.

The CNS indirectly modulates NMD by varying the neural drive to a muscle; a higher neural drive is directly associated to higher force output^6^, while the higher force output is associated with altered mechanical properties such as higher muscle stiffness^7^. NMD was shown to be modulated as a function of contraction speed, with shorter NMD associated with higher rate of force development^4,8^. Moreover, factors such as pain sensation can elongate NMD^8^. These findings have been derived from studies involving planned force modulation during sinusoidal feedback tasks, where the force is continually changing and are limited to relatively low mean contraction intensities of 10% and 20% of individuals’ maximal force^4,8^. In contrast, steady-force contractions, as part of classic trapezoidal force tasks, are utilized in the majority of studies employing MU detection^9–11^. During these contractions it is also possible to measure NMD due to the natural force fluctuation that occurs when the force level is intended to remain constant^12,13^.

NMD can vary considerably between muscle groups. In the first dorsal interosseous muscle, NMD ranges from approximately 100 to 300 ms, whereas in the tibialis anterior, it varies between 200 and 450 ms, depending on contraction speed and measured at low contraction intensities (10 and 20% MVC) during modulated contractions^4,8^. During steady-force contractions, the tibialis anterior has been shown to exhibit an NMD of ~ 150 ms^13^ and the triceps surae of ~ 500 ms^12^. However, interpreting absolute NMD values requires careful consideration of the specific task (e.g., fast/slow modulated vs. steady-force contractions), the muscle group studied, and the different types of dynamometers used^12^.

Martinez-Valdes et al.^13^ identified a relationship between fascicle length and NMD during steady-force contractions. They modulated fascicle length in the tibialis anterior by changing the ankle angle from neutral (0°) to plantar-flexed (30°) and found that shorter fascicle lengths resulted in shorter NMD at the same relative contraction intensity^13^. Furthermore, it has been suggested that NMD at low to moderate intensities, such as 20 and 40% MVC in the tibialis anterior^13^ and 10 and 40% MVC in the triceps surae^12^, does not differ significantly between intensities. Contreras-Hernandez et al.^12^ reported an 8% reduction in absolute NMD in the triceps surae, from 500 (± 120) to 460 (± 135) ms from 10 to 40% MVC. However, the standard deviations at both force levels exceeded the absolute reduction in NMD, which likely contributed to the lack of statistical significance. Importantly, these studies did not examine contraction intensities greater than 40% MVC. Therefore, the behavior of NMD across a broad spectrum of contraction intensities, from low to high muscle forces, remains unresolved.

The vastus lateralis (VL) and medialis (VM), as compartments of the quadriceps muscle, are pure extensors of the knee joint and play a crucial role in leg extension and functional movements such as walking, running, and maintaining posture. In this study, the neural drive to the quadriceps was assessed as the summed MU firings of VL and VM described by the cumulative spike train (CST). The CST reflects the summed binary information (firing or not firing) of the analyzed MUs. Accumulating neural information from muscle compartments within the same muscle is an established approach to describe overall neural drive to multi-compartment muscle^12,14–16^. Recent findings also confirm that VL and VM motor units receive common input by the CNS during voluntary contractions^17^. VL and VM share a larger proportion of common input compared to synergistic hand muscles^18^ or calf muscles, while ~ 73% of VL and VM motor unit spike trains are positively correlated with each other during a contraction (but only 22–28% of triceps surae muscles)^19^.

This study aims to investigate how NMD of the quadriceps muscle behaves across different contraction intensities during steady-force contractions. We hypothesize that NMD shortens with increasing contraction intensities during steady-force tasks, analogous to the observed reduction in NMD with increasing rates of force development^4,8^. As force increases during a contraction, motor units are progressively recruited, resulting in a larger overall neural drive. Higher muscle force is achieved through both the recruitment of additional MUs and increased discharge rates per MU^20^ and associated with an increased muscle–tendon unit stiffness^7^. Faster contractions involve more rapid recruitment of additional MUs and higher discharge rates per MU^21^ and are associated with reduced NMD^4,8^. A similar reduction in NMD may therefore also occur during steady contractions at higher intensities, where neural drive is elevated through both greater MU recruitment and increased discharge rates.

## Methods

### Participants

Thirteen recreationally trained males (age 24.3 ± 2.8 years, height 180.4 ± 4.0 cm, body mass 79.8 ± 7.9 kg) volunteered for this study. Exclusion criteria were (1) injury or neurological or orthopedic disorders of the lower limb during the past year, (2) lower limb resistance training within 24 h before testing, and (3) left-footedness, due to substantial force control differences associated with footedness^22^. All participants provided written informed consent. The study protocol and methods were approved by our Institutional Review Board (approval-No. DT2024003) and performed in accordance with the Declaration of Helsinki standards.

### Task

**Fig. 1:**
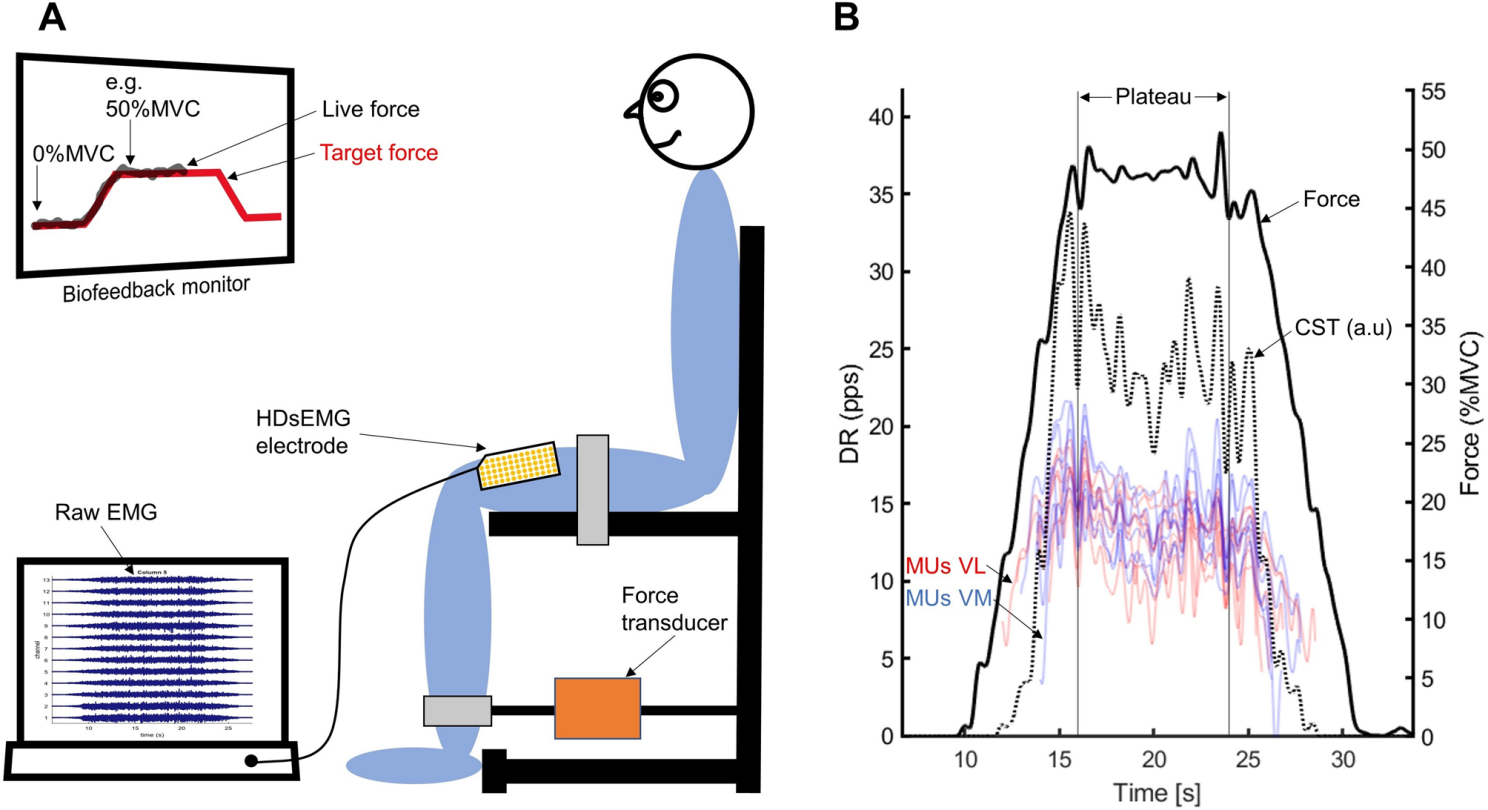
Illustration of the test setup. (**A**) Participant is placed in front of the biofeedback monitor (~ 1.5 m eye-to-screen distance) and fixed in position with a horizontal thigh and vertical shank. Measured force and target force are represented in real time and high-density surface electromyography (HDsEMG) recorded simultaneously. (**B**) Exemplary trial with 50%MVC target plateau force. Colored lines represent the filtered (2 Hz low-pass) discharge rate (DR) of motor units of the vastus lateralis (red) and the vastus medialis (blue). The cumulative spike train (CST) as the summed and filtered information of all detected motor units is shown as dashed line and the filtered force signal as solid black line. The plateau phase represents the middle 8 s of the steady-force phase of the trapezoidal task.

The participants performed isometric trapezoidal contractions while seated and fixed in position (Fig. 1A). Their right leg was positioned at a 90° knee angle (thigh horizontal, shank vertical). A strap was placed above the malleolus, connected to a force transducer (KAP-E, A.S.T, Germany), aligned at 90° to the shank. The force transducer was connected to an AD board (NI6218, National Instruments, USA) for force signal representation using a custom biofeedback tool (Matlab Version 2023b, MathWorks, USA).

Participants initially underwent a warm-up consisting of isometric contractions at 25%, 50%, and 75% of their perceived maximum force (held for 3–5 s, 1-minute rest intervals, twice per intensity). Participants then performed three maximum voluntary isometric contractions (MVCs) lasting 3–5 s each, separated by 2-minute rest intervals, with verbal encouragement provided and the live force curve displayed on a biofeedback monitor (~ 1.5 m eye-to-screen distance) in front of the participants. The highest force value reached among the three trials was taken as the MVC. Subsequently, participants completed trapezoidal biofeedback trials at steady target forces of 10%, 30%, 50%, and 70% of their individual MVC, while high-density surface electromyography (HDsEMG) signals were recorded. During these trials the target force was provided as a red line on the biofeedback monitor alongside the live force as a black line. The current live force was visualized in the middle of the horizontal axis of the screen while the target line was moving from the right to the left side of the screen (Fig. 1A). The participants were asked to reproduce the target line as precisely as possible with their applied force. The respective trapezoidal trial consisted of an ascending phase with an incline of 10%MVC/s, a 10-second steady-force phase, and a descending phase with a decline of 10%MVC/s. The trial order was randomized (2-minute rest intervals between trials).

### High-density electromyography

The electrode sites were shaved and cleaned with alcohol. The muscle fascicle direction of VL and VM was determined using guidelines for detecting muscle innervation zones^23^. The main innervation zones of VL and VM were identified with a 1-dimensional, non-adhesive 16-channel array electrode (10 mm inter-electrode distance) while the participant performed an isometric contraction at low force at ~ 10–20% of perceived maximum^24^. Muscle fascicle direction and main innervation zones were marked on the skin. For VL and VM, a separate 2-dimensional 64-channel electrode (GR08MM1305, OT Bioelettronica, Torino, Italy; 5 columns, 13 rows, 8 mm inter-electrode distance) was placed on each muscle. The rows were aligned with the muscle fascicle direction, and the main innervation zone was positioned between the 6th and 7th row. Electrodes were connected to the amplifier (Quattrocento, OT Bioelettronica, Italy; 16-bit) and recorded with the OT Biolab + software (v1.5.9, OT Bioelettronica, Italy) in monopolar mode at 2048 Hz sample rate. Electrode channels with baseline electric noise < 40 µV were accepted, those with higher noise were excluded from further processing^25^.

### Data processing

The middle 8 s of the 10-second steady-force phase were selected for further analysis and referred to as plateau from here on (Fig. 1B). The HDsEMG signals were digitally filtered using a 2nd order zero-phase Butterworth band-pass filter (20–500 Hz) and decomposed into MU spike trains using the DEMUSE software (Maribor University, Maribor, Slovenia). DEMUSE is a reliable decomposition tool^11^ incorporating a validated decomposition algorithm based on Convolution Kernel Compensation^26–29^. Each frame of a MU spike train contains binary information, with a value of 1 indicating that the MU is firing and 0 indicating that it is not firing. All MU spike trains were manually inspected and corrected if necessary^25^. MU spike trains with a pulse-to-noise ratio (PNR) < 30 db were excluded from further analysis^27^. The instantaneous discharge rate (IDR) for MUs with a PNR ≥ 30db was calculated as the inverse of the inter-spike time interval (e.g. an inter-spike interval of 90 ms leads to an IDR of 1/0.09 s = 11.11 pulses per second (pps)). MUs with an inter-spike interval above 250 ms during the plateau were discarded from analysis. The mean MU discharge rate was calculated as the mean of the IDR during the plateau. The MU recruitment threshold was defined as the force level, expressed in %MVC, at which the first firing of the individual MU occurred during the ascending phase.

### Neuromechanical delay

**Fig. 2:**
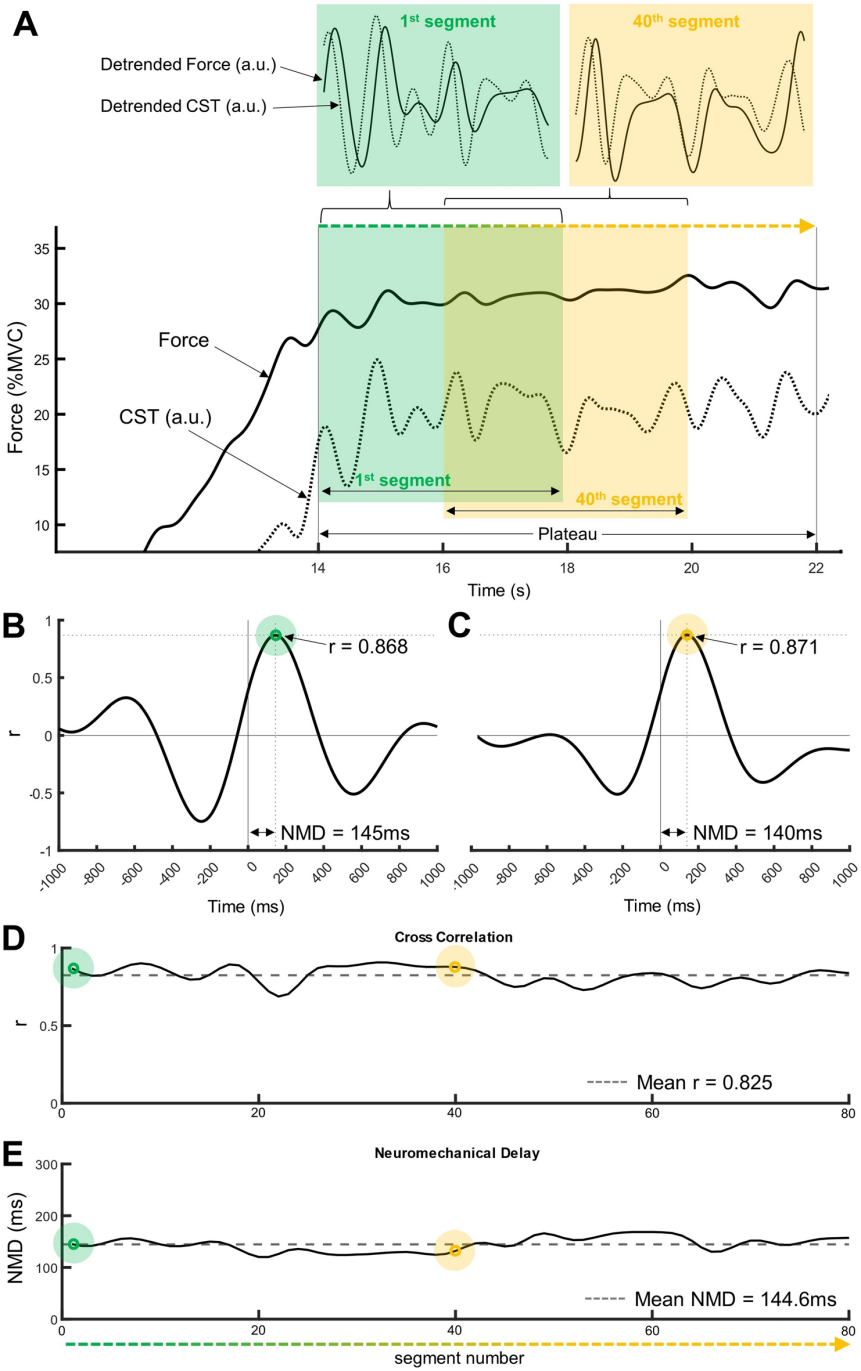
Calculation of time lag between neural drive and force. (**A**) Force and cumulative spike train (CST), both 2 Hz low-pass filtered, from an exemplary trial with a 30%MVC plateau target force. During the plateau, 4-second analysis segments beginning at the start of the plateau (green, 1st segment of 80) and moving in 50ms steps toward the end of the plateau (e.g. yellow, 40th segment of 80) are assessed. Detrended force and CST (see small figures on the top) were cross-correlated for each segment; note that the force curve follows the neural drive curve with a time delay. (**B**) The cross-correlation function for the 1st segment and (**C**) 40th segment each reveal a different maximal cross-correlation coefficient and corresponding time lag (neuromechanical delay (NMD)). (**D**) Maximal cross-correlation and (**E**) corresponding NMD for the 80 segments. Mean cross-correlation coefficient and mean NMD (dashed lines) across the 80 segments were analyzed further.

In order to assess the neural drive during the plateau of each trial, MU spike trains of VL and VM were summed to generate the cumulative spike train (CST). CSTs containing at least 2 MUs from both VL and VM were included in the analysis, resulting in each CST comprising 4 or more MU spike trains. Two routines for CST and force filtering were conducted. The first routine is an established filter which addresses low frequency fluctuation within the CST and force^4,8,12,13^; the second routine is a recently recommended estimation including higher frequencies^30^. First routine: CST and submaximal force signal were smoothed using a 4th -order zero-phase Butterworth 2 Hz low-pass filter to extract only low-frequency oscillations^4^ and detrended with a 4th -order zero-phase Butterworth 0.75 Hz high-pass filter to remove very slow variations^31^ (Fig. 2A). These filtering steps were presented previously for modulated^4,8^ and steady-force contractions^12,13^ in order to estimate NMD. Second routine: According to Tvrdy et al.^30^ the optimal filtering routine for steady contractions involves the smoothing of CST and submaximal force with a 400 ms Hanning window, followed by a 0.75–5 Hz 2nd -order zero-phase Butterworth bandpass filter. For the filtered CST and submaximal force revealed from each routine, the following analysis was conducted. Consecutive cross-correlation functions for 4-second segments of the plateau were calculated for the detrended force and CST (Fig. 2A). The first 4-second segment began at the start of the plateau, with each subsequent segment starting 50 ms after the beginning of the previous one, resulting in 80 cross-correlograms (Fig. 2B/C). The peak cross-correlation coefficient and the corresponding time lag (i.e. NMD) of each cross-correlogram were identified for each segment (Fig. 2D/E). The small step size (50 ms) and large overlap of the segments allow an accurate estimation of the mean NMD and mean cross-correlation at a high temporal resolution. The mean NMD and mean cross-correlation coefficient of the 80 segments for each participant and contraction intensity were analyzed further.

### Statistical analysis

A two-way ANOVA was conducted to examine the effects of muscle (factor 1, two levels: VL and VM) and contraction intensity (factor 2, four levels: 10, 30, 50, and 70%MVC) on MU discharge rate and recruitment threshold (for this analysis the thirteen participants’ MUs were pooled together). A linear mixed-model analysis was performed to assess the effect of contraction intensity (four levels: 10, 30, 50, and 70%MVC) on the mean NMD and cross-correlation coefficient. The Bonferroni-Holm correction was applied to adjust for multiple comparisons, with the significance level set at *p* < 0.05 for all statistical tests. Statistical analyses were performed using JASP (Version 0.19.1, JASP Team, Netherlands).

## Results

### Force

The thirteen participants produced a mean MVC (± SD) of 704.9 ± 167.0 N with a range of 447.1 to 928.6 N.

### Motor unit characteristics

**Fig. 3:**
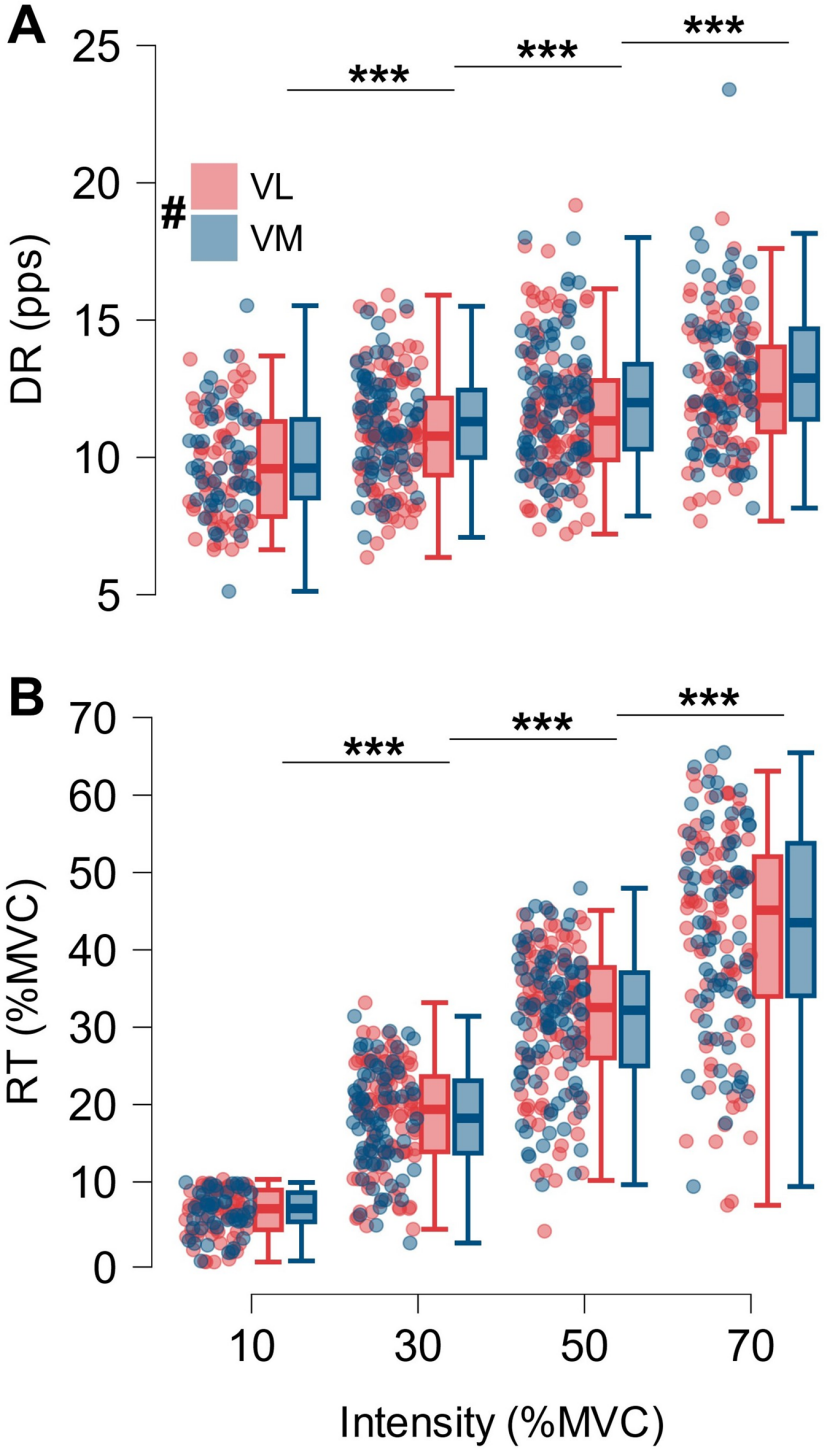
Motor unit discharge characteristics. (**A**) Box plots of mean discharge rate (DR) during the plateau and (**B**) motor units’ recruitment threshold (RT) per muscle (VL red, VM blue) and across intensities. Motor units of all participants were pooled together while each dot represents a motor unit. # indicates a significant main effect of muscle. Significant post-hoc comparisons between intensities: *** *p* < 0.001.

A total of 653 MUs were analyzed (VL: 374, VM: 279). MU counts per muscle and intensity across participants are presented in Table 1. For two subjects, no MUs could be identified in the VL at 70%MVC. Mean MU discharge rate during the plateau increased with contraction intensity (F_(3,649)_ = 44.81, *p* < 0.001, ω² = 0.167), for the VL from 9.7 ± 1.5 pps at 10%MVC to 11.0 ± 1.4 pps at 30%MVC, 11.9 ± 2.0 pps at 50%MVC, and 12.1 ± 1.8 pps at 70%MVC and for the VM from 10.2 ± 2.4 pps at 10%MVC to 11.4 ± 1.2 pps at 30%MVC, 12.3 ± 1.8 pps at 50%MVC, and 13.5 ± 2.4 pps at 70%MVC, with significant differences between all intensities (Fig. 3A). There was a significant main effect of muscle, although with a small effect size (F_(1,649)_ = 5.73, *p* = 0.017, ω² = 0.006). Mean MU recruitment threshold increased with contraction intensity (F_(3,649)_ = 412.32, *p* < 0.001, ω² = 0.655), for the VL from 6.0 ± 1.9%MVC at 10%MVC to 18.8 ± 3.8%MVC at 30%MVC, 31.5 ± 3.5%MVC at 50%MVC, and 44.9 ± 8.9%MVC at 70%MVC and for the VM from 5.5 ± 2.3%MVC at 10%MVC to 18.5 ± 4.5%MVC at 30%MVC, 30.2 ± 6.8%MVC at 50%MVC, and 44.7 ± 11.5%MVC at 70%MVC, with significant differences between all intensities (Fig. 3B). At a constant ramp slope, the same motor unit is expected to exhibit a similar recruitment threshold across trials^32^. Due to the variation of MUs detected at distinct recruitment thresholds at the different intensities, we did not track whether MUs were reappearing across the contraction intensities. As shown in Fig. 3B, all MUs at 10% MVC target force were recruited within the 0–10% MVC range, whereas at 70% MVC, only a small proportion of MUs were recruited in this low-force range. This methodological phenomenon is further discussed in the limitations section.

**Table 1:** Mean ± standard deviation (SD) and range of the number of motor units detected and used for analysis for each muscle and contraction intensity per participant (*n* = 13).

Intensity (%MVC)	M. vastus lateralis	M. vastus medialis
	mean ± SD (range)	
10	5.3 ± 2.7 (2–10)	3.7 ± 2.6 (1–9)
30	8.1 ± 2.8 (2–12)	5.7 ± 5.7 (2–11)
50	8.4 ± 3.2 (3–13)	6.8 ± 4.5 (1–13)
70	7.0 ± 4.6 (0–12)	5.3 ± 3.9 (1–13)

### Neuromechanical delay

**Fig. 4:**
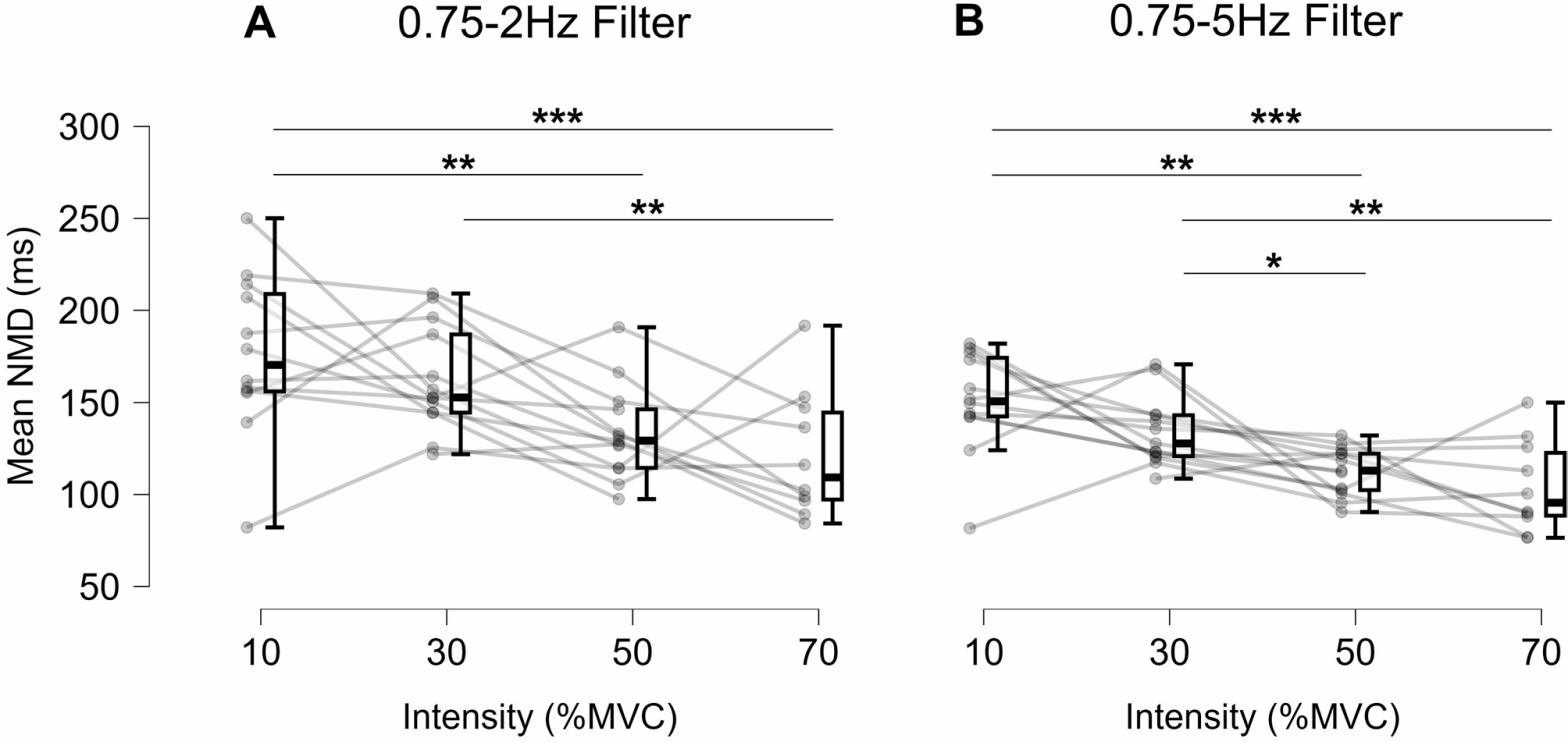
Neuromechanical delay results. Box plots of mean neuromechanical delay (NMD) across intensities for the two filtering routines: 0.75–2 Hz filter^4,8,12,13^ (**A**) and 0.75–5 Hz filter^30^ (**B**). Grey dots represent participants’ individual data. Significant post-hoc comparisons: * *p* < 0.05 ** *p* < 0.01 *** *p* < 0.001.

One participant did not meet the criterion of detecting at least two motor units per muscle at 10%MVC, and three participants did not meet it at 70%MVC. To address this, a linear mixed-model analysis was conducted to estimate the effect of contraction intensity on mean NMD and the cross-correlation coefficient. The NMD and cross-correlation calculations were applied for two separate filter routines: (1) 0.75–2 Hz filter^4,8,12,13^ and (2) 0.75–5 Hz filter^30^. The 0.75–2 Hz filter routine revealed that the mean NMD decreased as contraction intensity increased, from 175.8 ± 44.1 ms at 10%MVC to 162.5 ± 28.7 ms at 30%MVC, 133.4 ± 25.4 ms at 50%MVC, and 121.6 ± 34.6 ms at 70%MVC, with a significant effect of contraction intensity (F_(3,33.57)_ = 7.83, *p* < 0.001, ω² = 0.359). Post-hoc comparisons showed significant differences between 10% and 50%MVC (*p* < 0.01), 10% and 70%MVC (*p* < 0.001), and 30% and 70%MVC (*p* < 0.01) (Fig. 4A). The mean cross-correlation coefficient was 0.62 ± 0.15, 0.73 ± 0.07, 0.69 ± 0.09, and 0.73 ± 0.18 for 10, 30, 50, and 70%MVC, with a main effect of contraction intensity (F_(3,32.59)_ = 3.04, *p* = 0.043, ω² = 0.147), and significant post-hoc contrast between 10% and 30%MVC (*p* = 0.047). The 0.75–5 Hz filter routine revealed that the mean NMD decreased as contraction intensity increased, from 150.4 ± 28.1 ms at 10%MVC to 134.0 ± 18.9 ms at 30%MVC, 112.6 ± 13.2 ms at 50%MVC, and 104.3 ± 24.9 ms at 70%MVC, with a significant effect of contraction intensity (F_(3,44)_ = 10.66, *p* < 0.001, ω² = 0.392). Post-hoc comparisons showed significant differences between 10% and 50%MVC (*p* < 0.01), 10% and 70%MVC (*p* < 0.001), 30% and 50%MVC (*p* < 0.05), and 30% and 70%MVC (*p* < 0.01) (Fig. 4B). The mean cross-correlation coefficient was 0.62 ± 0.14, 0.69 ± 0.07, 0.65 ± 0.09, and 0.69 ± 0.14 for 10, 30, 50, and 70%MVC, without an effect of contraction intensity (F_(3,32.74)_ = 1.60, *p* = 0.208, ω² = 0.051).

## Discussion

This study provides an investigation of neuromechanical delay (NMD) during steady-force contractions, offering novel insights into the time dependency on neural drive, as NMD decreases with increasing contraction intensity. The NMD, estimated as the time lag between neural and force fluctuations, decreased by 31%, from 176 to 122 ms (0.75–2 Hz filter) and 150 to 104 ms (0.75–5 Hz filter) when moving from 10 to 70% of MVC. This shorter NMD with higher intensities indicates a faster coupling between neural drive and motor output, suggesting that underlying mechanisms such as MU recruitment play key roles in this process by influencing mechanical muscle properties.

To assess NMD, we applied two separate filtering routines used in previous NMD studies: (1) a 2 Hz low-pass filter for smoothing and a 0.75 Hz high-pass filter for detrending the neural drive and force signal^4,8,12,13^ and (2) a 400ms Hanning window for smoothing and a 0.75–5 Hz bandpass filter as proposed by Tvrdy et al.^30^, followed by a cross-correlation analysis. For the estimation of the mean NMD, we calculated the average of subsequent 4-second segments, each starting 50 ms after the beginning of the previous one, resulting in a total of 80 segments across the 8-second plateau phase (Fig. 2). This approach allows for a more detailed resolution of NMD estimation compared to 5-second segments with 50% overlap used in previous studies^12,13^. In our study, the mean cross-correlation of neural drive and force fluctuation of the quadriceps across the four intensities was on average ~ 0.70 using the 0.75–2 Hz filter, which is slightly higher than previously reported for the triceps surae (~ 0.55^15^ and ~ 0.68^12^) and slightly below the findings of the tibialis anterior (~ 0.70^13^ and ~ 0.76^4^) and for the first dorsal interosseus (~ 0.83^4^). The average cross-correlation coefficient of ~ 0.70 (0.75–2 Hz filter) and ~ 0.65 (0.75–5 Hz filter) indicates a robust estimation of NMD of the quadriceps in our study. The slightly lower cross-correlation coefficient of CST and submaximal force when using the 0.75–5 Hz filter compared to the low frequency bandwidth (0.75–2 Hz filter) was reported previously and is considered as the consequence of over-smoothing the signals when applying the low frequency filter^30^.

However, the choice of filter did not alter the overall force-dependent decrease in NMD. Notably, applying the 0.75–5 Hz filter revealed an additional statistically significant reduction (*p* < 0.05) between the 30% and 50%MVC contraction intensities (Fig. 4). This filter also resulted in lower absolute NMD values and reduced data variability.

The reduction in NMD with increased contraction intensity might be partially explained by the recruitment of higher-threshold MUs. Spike trains of single motor units with higher recruitment thresholds are associated with shorter delays to the corresponding force output^33^. As contraction intensity rises, additional larger and stronger motor units are recruited, characterized by faster muscle fiber conduction velocity^34^, shorter time to peak of muscle force^35^, increased force and power production capacity due to larger cross-sectional area and myosin heavy chain isoform composition^36^. Thus, the involvement of higher-threshold MUs is likely influencing these mechanical properties of the muscle, contributing to a reduction in NMD. Our findings confirm the involvement of higher-threshold MUs at higher forces since mean MU recruitment threshold increased substantially with rising contractions intensity (Fig. 3B).

While it was demonstrated that NMD shortens with shorter fascicle length provoked through different joint angles but at same relative contractions intensity^13^, it is also known that the vastus lateralis fascicle length shortens with increasing contraction intensity during isometric contractions at a fixed knee angle^37^. Shorter fascicle length and increased muscle stiffness at higher contraction forces may result in a stiffer muscle-tendon unit, thereby enhancing the efficiency of force transduction from the muscle to the skeletal system^7^ possibly contributing to the NMD reduction as seen in our data. Despite being plausible, this remains speculative as we did not track fascicle length or muscle-tendon unit stiffness in our study.

Contrary to previous studies that reported no significant differences in NMD at low to moderate contraction intensities, our findings demonstrate a significant effect of contraction intensity on NMD when higher force levels are considered. Martinez-Valdes et al.^13^ observed that NMD did not differ significantly between 20% and 40% MVC in the tibialis anterior. Similarly, Contreras-Hernandez et al.^12^ reported an 8% reduction in absolute NMD in the triceps surae from 500 (± 120) ms at 10% MVC to 460 (± 135) ms at 40% MVC, which lacked statistical significance, likely due to the large standard deviations exceeding the observed NMD reduction. Notably, these studies did not investigate contraction intensities greater than 40% MVC. In our study, if we had limited our analysis to 10% and 30% MVC, we would have observed a 7.4% reduction in NMD, from 176 ms to 163 ms using the 0.75–2 Hz filter, but without reaching statistical significance. By including higher contraction intensities of 50% and 70% MVC, we were able to investigate the behavior of NMD across a broader range of forces. This approach demonstrated a significant effect of contraction intensity on NMD, highlighting the importance of examining higher force levels to capture meaningful changes in NMD.

Unlike studies that focus on planned force modulation during isometric contractions^4,8^, our approach involved maintaining a constant isotonic-isometric contraction. This steady-force paradigm simplifies the neuromuscular control requirements for the CNS, as it does not require continuous adjustments in motor unit recruitment and discharge rate throughout the contraction. By focusing on steady-force contractions, we provide a basic understanding of neuromechanical coupling under conditions where force output is held constant, which might also be interesting in settings beyond basic human research. Nuccio et al.^38–40^ showed that patients have altered neural drive and force control of the quadriceps muscle after anterior cruciate ligament reconstruction. Hence, analyzing NMD during steady-force contractions may help provide useful insights into the rehabilitation status of such patients, for example in terms of their progress over time or comparing the injured and non-injured leg. In addition, NMD could also be used to possibly extend our understanding of muscle fatigue since it has been described that neural drive processes during prolonged contractions are related to task failure^41^.

### Limitations

Studies using HDsEMG to identify MU firing patterns face the challenge that the detected MUs do not represent the entire active MU pool, potentially leading to an over-representation of higher-threshold MUs and an under-representation of lower-threshold MUs^6^. Focusing on the range of MUs recruitment threshold, it is notable that the number of detectable low-threshold MUs (i.e., those recruited before 10%MVC) decreased at higher intensities (Fig. 3B). However, since the twitch force of a MU increases with increasing recruitment threshold^35^ and higher-threshold MUs produce substantially more force^36^, we assume that higher-threshold MUs contribute to a larger extent to overall muscle force, still leading to a robust estimation of neural drive and NMD. Additionally, the findings of this study are restricted to healthy young males. Future research is necessary to confirm these results in females and to explore potential variations across different age groups.

## Conclusion

This study investigated neuromechanical delay during steady-force contractions, highlighting its dependency on contraction intensity. The findings reveal that NMD during natural force fluctuations decreases with increasing contraction intensity, suggesting a faster coupling between neural drive and motor output at higher forces. This likely reflects changes in muscle mechanical properties due to higher-threshold motor unit recruitment. These findings enhance our understanding of the time dependency between neural drive and motor output across a broad range of contraction intensities.

## Data Availability

The datasets generated and analyzed during this study are available from the corresponding author on reasonable request.
